# Molecular mechanism(s) of endocrine-disrupting chemicals and their potent oestrogenicity in diverse cells and tissues that express oestrogen receptors

**DOI:** 10.1111/j.1582-4934.2012.01649.x

**Published:** 2012-12-20

**Authors:** Hye-Rim Lee, Eui-Bae Jeung, Myung-Haing Cho, Tae-Hee Kim, Peter C K Leung, Kyung-Chul Choi

**Affiliations:** a Laboratory of Veterinary Biochemistry and Immunology, College of Veterinary Medicine, Chungbuk National UniversityCheongju, Chungbuk, Korea; b Laboratory of Toxicology, College of Veterinary Medicine, Seoul National UniversitySeoul, Korea; c Advanced Institute of Convergence Technology, Seoul National University, SuwonKorea; d Department of Obstetrics and Gynecology, College of Medicine, Soonchunhyang UniversityBucheon, Korea; e Department of Obstetrics and Gynecology, Child and Family Research Institute, Faculty of Medicine, University of British ColumbiaVancouver, British Columbia, Canada

**Keywords:** endocrine-disrupting chemicals, oestrogen receptor, oestrogenicity

## Abstract

Endocrine-disrupting chemicals (EDCs) are natural or synthetic compounds present in the environment which can interfere with hormone synthesis and normal physiological functions of male and female reproductive organs. Most EDCs tend to bind to steroid hormone receptors including the oestrogen receptor (ER), progesterone receptor (PR) and androgen receptor (AR). As EDCs disrupt the actions of endogenous hormones, they may induce abnormal reproduction, stimulation of cancer growth, dysfunction of neuronal and immune system. Although EDCs represent a significant public health concern, there are no standard methods to determine effect of EDCs on human beings. The mechanisms underlying adverse actions of EDC exposure are not clearly understood. In this review, we highlighted the toxicology of EDCs and its effect on human health, including reproductive development in males and females as shown in *in vitro* and *in vivo* models. In addition, this review brings attention to the toxicity of EDCs *via* interaction of genomic and non-genomic signalling pathways through hormone receptors.

- Introduction
  – Biological function of oestrogen and its receptors
  – Oestrogenic actions of EDCs via ER-mediated signalling
  – Detrimental effect of various EDCs
  – Effects of EDCs on human health
  – Novel assays to evaluate EDCs
- Conclusion

## Introduction

Various endocrine-disrupting chemicals (EDCs) are found in the environment. These EDCs interfere with the regulation of hormone synthesis or receptor binding by altering the hormone homeostasis of endocrine system [1, 2]. In doing this, EDCs can cause reproductive, development and sexual behaviour dysfunction and lead to detrimental results in animals and human beings. Because almost EDCs from natural or synthetic sources have structures similar to those of endogenous steroid hormones including oestrogen (E2) or androgen, they tend to interfere with the actions of steroid hormones *via* binding to the corresponding hormone receptors. Oestrogen is involved in several mechanisms in mammals including not only reproduction but also bone integrity, adipogenesis and behaviour [3, 4]. Over the past 20 years, major attention has been given to the critical effects of EDCs which have been released in environment on human beings. Endocrine-disrupting chemicals are used widely in industry and found throughout the world, including plant constituents and pesticides. Exposure of EDCs may develop serious abnormalities including impaired reproductive function and formation of several hormone-dependent cancers such as breast and ovarian cancer in women and children [5, 6].

Many synthetic chemicals and natural plant compounds are known as xeno-oestrogen which bind to the oestrogen receptor (ER) with an affinity 1000-fold lower than that of oestrogen. These EDCs appear to induce tissue-specific oestrogenic responses as an ER agonist or antagonist, resulting in dysregulation of ERα-dependent transcriptional signalling pathways [7, 8]. In addition, suspected environmental oestrogenic EDCs have been used as biological reagents or drugs to treat hormone-related disorders in human beings. For example, diethylstilbestrol (DES) was prescribed to block spontaneous abortion along with medications for preventing miscarriage between the 1940s and 1970s [9]. However, DES is known as a carcinogen in human beings [10] and increases the risk of breast cancer in mothers and daughters exposed to DES during pregnancy [11]. This compound is a non-steroid oestrogen which can mimic oestrogenic actions *via* an ER-signalling pathway [10, 12].

In addition to DES, man-made synthetic EDCs of pesticides and chemicals used for plastic manufacturing, including dichlorodiphenyltrichloroethane (DDT), bisphenol A (BPA), octyl-phenol (OP), nonyl-phenol (NP) and methoxychlor (MXC), can pass through the placenta to the foetus as shown in previous studies [13]. These EDCs have a sufficient affinity to steroid hormone receptors, *i.e*. ER, progesterone receptors (PRs) and/or androgen receptor (AR), which alter hormone receptor responsiveness [14]. However, recent studies indicated that the effects of OP, NP and BPA exposure on the induction of non-genomic pathways have been observed in the cells. EDCs induced an alternative mechanism related with the activation of ERK1/2, Akt1/2/3 and/or G-proteins [15, 16]. Recent studies have shown that the deregulated activation of other signalling pathway by EDCs involves ER-mediated signalling interactions with transduction of signalling cascade regulated by phosphorylation [17].

Endocrine-disrupting chemicals can cause severe dysfunction of endocrine, reproductive and developmental systems in both males and females. In particular, EDCs are thought to be associated with reproduction abnormalities in animals and human beings, although the precise effects on human health are still not clear [18]. Normal function and maturation of the sexual reproductive glands and tract are affected by EDCs during the development process. Endocrine-disrupting chemicals may also stimulate cancer growth and exert toxic effects on the neuronal and immune systems. Consequently, EDCs are suspected to be potentially dangerous chemicals with global concern, but only a few *in vitro* and *in vivo* assays have been developed to determine whether a chemical have potency to disrupt endocrine system or not. Most data from *in vitro* and *in vivo* assays are derived by measuring oestrogenic and androgenic activity [19, 20].

This review describes the detrimental effects of several EDCs on human health including those specific for the reproduction, neuronal and immune systems. We also summarize the *in vitro* and *in vivo* assays used to detect EDCs. Finally, we focused our attention on a novel *in vivo* immature rat model which uses the induction of calbindin-D_9k_ (CaBP-9k) mRNA and protein as a biomarker for detection of EDCs [21–24].

### Biological function of oestrogen and its receptors

E2 is a major steroid hormone which is important for regulating diverse physiological sexual behaviour functions, for instance, reproductive organ development, bone formation and bone remodelling, cardiovascular regulation and the modulation of inflammation [25]. This steroid hormone is thought to be important for the development of secondary sexual characteristics and sexual behaviour, regulation of hypothalamic expression and release of gonadotropin-releasing hormone (GnRH) in human beings and mammals [26]. However, two gonadotropin hormones, luteinizing hormone (LH) and follicle stimulating hormone (FSH), control the production of oestrogen in ovulating women [27]. Oestrogen is mainly derived from its synthesis in the theca cells in the ovarian follicle. In addition, oestrogen is produced by the corpus luteum in the ovary and the placenta. Recent studies have suggested that the liver, adrenal glands and mammary glands may also contribute to the production of E2, although the quantity is insignificant [28, 29]. In rodents, oestrogen release is necessary for sexual responsiveness and facilitates the complex function of other sex hormones in males and females [30]. Oestrogen exists in men as well as women with E2 contributing to the differentiation and function of Leydig cells and development of testes in males [31].

The hypothalamic-pituitary-gonadal (HPG) axis consists of GnRH neurons of the hypothalamus, gonadotropes in the anterior pituitary gland and somatic cells in the gonads [32]. Somatic cells in the gonads include not only theca cells and granulosa cells in the ovary but also Leydig and Sertoli cells in the testis. The anterior lobe contains hormone-producing cells and supports folliculo-stellate cells; the anterior pituitary gland secretes gonadotropins including LH and FSH [33]. The intermediate lobe is composed of primarily melanotrophs whereas the neural lobe is made up of pituicytes and nerve endings. Diverse pituitary cells are known target cells of oestrogen, including lactotrophs and gonadotrophs [34].

E2 regulates cell function through specific, tissue-dependent, intracellular responses and can stimulate the activation of oestrogen-dependent metabolism. The activation of the ERs by E2 binding is associated with the expression of many related genes through strong interaction with an oestrogen response element (ERE) in the promoter [35, 36]. Previous studies demonstrated that ERs are localized in diverse intracellular spaces. After E2 binds to ERs, the ERs undergo a conformational change resulting in assembly and interaction with cofactor molecules, coactivators and corepressors related to gene expression [37]. These ligand-receptor complexes can bind to EREs in promoter regions of the target genes to control either activation and/or repression of gene expression [38]. In addition, interaction between membrane ERs and nuclear ERs is needed for signalling cascade integration and activation of secondary messengers by receptor tyrosine kinases such as the epidermal growth factor receptor (EGFR) [39, 40]. Nuclear ERs-mediated transcriptional activation, referred to as genomic pathway, requires several hours for establishment whereas membrane ERs can activate ligand-independent pathways, referred to as non-genomic pathways, within minutes after exposure of ligands [41, 42].

Membrane ERs are important for cell function modulation through non-genomic pathways, which results from phosphorylation *via* crosstalk between the membrane ER and other signalling pathways [43]. Although there is increasing evidence that different signalling pathway are induced by E2, the precise relation between ER and E2 remains to be elucidated [44]. The conformational changes in the ER lead to the dissociation of the chaperone and formation of the dimerized ERs, which can stimulate DNA binding and facilitate the interaction between coregulators and transcriptional machinery [45].

Oestrogen receptor-α and ER-β have distinct molecular mechanisms and distribution on oestrogen-dependent specific tissue. ER-α was first investigated in the 1960s and was isolated from MCF-7 cells in 1986 [46]. ER-β was discovered and cloned from rat prostate 10 years later [47]. The human ER-α gene is localized on chromosome 6 and the ER*-*β gene is on chromosome 14 [48]. The ER-α protein consists of 595 amino acids and has a molecular mass 66 kD; the ER-β protein has approximately 530 amino acids and a molecular mass 54 kD [49]. Transgenic knockout mice studies suggested that ER-α is more important in the uterus and female mammary glands, but ER-β is found primarily in the ovary and the prostate gland. ER-α activates gene transcription in the presence of E2 while ER-β inhibits the expression of activator protein 1 (AP-1) [50], an ubiquitous transcription factor consisting of c-Jun and c-Fos. ER-α and ER-β have opposite effects on cyclin D1 regulation. Cyclin D1 levels are increased by ER-α and are decreased by ER-β [51]. ER-β has been known to contribute to apoptosis and the regulation cell proliferation in ovarian cancer [52]. As previously noted, transcriptional activation of ER-α and ER-β is different depending on the promoter, ligand affinity and cell type [53].

### Oestrogenic actions of EDCs *via* ER-mediated signalling

**Fig. 1:**
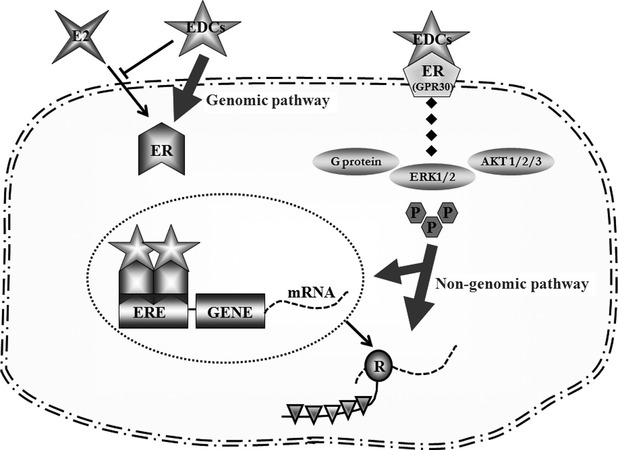
Potential mechanism(s) of endocrine-disrupting chemicals (EDC) action. In the ‘genomic pathway’ of EDC action, EDCs interfere with oestrogen (E2) binding to oestrogen receptors (ERs). EDCs bind to ERs instead of E2, and can thus affect the transcription of target genes in the nucleus by binding to the oestrogen response element (ERE) of target genes. The ‘non-genomic pathway’ of EDC action may occur through ER such as G protein-coupled receptor (GPR30) located in the cytoplasmic membrane. Activation of GPR30 by EDCs leads to rapid downstream cellular signalling. This induces subsequent stimulation of protein kinase activation and phosphorylation, which in turn may affect the transcription of target genes. The resulting changes by interaction between ERs and GPR30 in gene expression and intracellular signalling can cause cellular response without regulation, which may produce adverse effects of EDCs on organs.

Endocrine-disrupting chemicals are thought to act primarily through nuclear hormone receptors including ERs, ARs, PR, thyroid receptors (TRs) and others. Endocrine-disrupting chemicals include synthetic chemicals used as polychlorinated biphenyls (PCBs), polybrominated biphenyl, phthalates, BPA, NP, OP, DDT and DES [54]. Natural chemicals found in human and animal food including phyto-oestrogens (genistein and coumestrol) can also act as endocrine disruptors with oestrogen activity [55]. Although these substances are generally believed to have low binding affinity with ER-α and/or ER β, these chemicals are widely consumed in the world. Endocrine-disrupting chemicals often have a phenolic structure that enables these chemicals to act as endogenous hormones. Consequently, EDCs are able to interact with steroid hormone receptors as agonist or antagonists [14]. These compounds can also disrupt ERα-mediated transcriptional activity through crosstalk between the ERs and other nuclear receptors (NRs), and growth factor modulation [56]. Furthermore, EDCs can stimulate ERα-dependent kinase pathways through membrane ERα or G protein-coupled receptor (GPR30) [57, 58]. As previously described, EDCs can bind to ERs, and affect the transcription of target genes *via* genomic and/or non-genomic pathways. However, genomic pathway of oestrogen receptors is defined by modulation of transcriptional processes undergoing nuclear translocation and binding on ERE, on this, leading to regulation of target gene expression. On the other hand, non-genomic pathway passes signal transduction starting from steroid hormone receptors, which is distinct from response by ERs in cytosol (Fig. 1) [59]. These non-genomic signalling include the rapid pathway such as activation of second messengers or kinases, which is given a signal to stat through GPR30, the novel seven-transmembrane oestrogen receptor, which is structurally different to the ER, mediates rapid actions of 17 beta-estradiol and EDCs [60]. In this regard, recent studies have shown that GPR30 was involved in the proliferative effects induced by EDCs in both normal and cancer cells *in vivo* and *in vitro* [61]. Hence, GPR30 signalling pathway should be included among the signal transduction mechanisms through which EDCs may cause the abnormal oestrogen-related signalling response [62]. In addition, the oestrogenic activity of EDCs may be detrimental to human beings owing to altered gene expression or the effects on steroidgenic enzymes [63, 64]. Studies on the characterization of tissue-specific EDC oestrogenic activities are limited, as well as the methods for assessing their associated hazards and risks.

### Detrimental effect of various EDCs

Oestrogen is a sex hormone produced by ovaries and testes that is responsible for sexual development, normal functions and biosynthesis of nerve cells. Whereas oestrogen is beneficial and essential for the human health, synthetic substances like EDCs that mimic oestrogen have adverse effects. EDCs have been shown to affect the target cells in a dose-dependent manner, owing to their ability to bind ERs in both *in vitro* and *in vivo* [65, 66]. Reproductive abnormalities in animals exposed to EDCs can be observed and an increase of patients in hormonal-dependent cancers is a public concern.

#### Dichlorodiphenyltrichloroehane (DDT)

Dichlorodiphenyltrichloroethane (DDT) was the first general insecticide. The use of DDT was banned by many countries in the 1970s. The major metabolite of DDT, 1,1-dichloro-2,2-bis ethylene (DDE), inhibits prostaglandin synthesis in reptiles and birds, which results in weakened eggshells because of thinning. DDT binds the ER-α and induces transcriptional activity in ER-α-positive breast cancer MCF-7 cells [67]. This chemical is known to interference with endocrine system homeostasis in animals because of binding to the ER-α in both reproductive and other tissues after exposure and accumulation of DDT in the body. In addition, DDT activates ERs in the brain and liver of adult laboratory animals treated with high concentrations of DDT, resulting in the acute development of liver tumours [68]. However, the effects of DDT on ER-α signalling can be blocked by the anti-oestrogen ICI 182,780 [69]. Both DDT and DDE interfere with oestrogen biosynthesis; DDE also enhances aromatase activity. Aromatase is an enzyme important for regulating the conversion of androgens into oestrogens. The previous studies identified the effects of DDT and DDE on reproductive tissues in human beings [70, 71], but these findings have not been confirmed and require further study.

#### Bisphenol A

Bisphenol A is used as a plasticizer for the production of epoxy resins and polycarbonate plastics over the world. With the increasing use of plastics in industrialized societies, human exposure to BPA has increased in frequency [72]. BPA exposure during the perinatal period causes physiological and functional underdevelopment of both male and female genitalia, tracts and glands that may result in reduced fertility, aspermia, immature reproductive systems and the growth of several cancers such as breast, ovary and prostate cancer [73]. However, the binding affinity of BPA for ERs is approximately 1000-fold lower than that of E2, and BPA stimulates ER-α and ER-β signalling with oestrogen activity [74]. BPA promotes the proliferation of MCF-7 breast cancer cells both *in vitro* and *in vivo* [75]. This reagent can also increase PR expression in human endometrial, ovarian and breast cancer [76]. BPA activates ERE promoter constructs, which can be blocked by cotreatment with the ER-mediated signalling antagonist, ICI 182,780 [77]. In rodents, BPA induces vaginal phenotypic alteration, promotes early growth and differentiation of mammary glands and changes prolactin levels [78]. Recent studies have demonstrated that BPA increases uterine wet weight in rodents [79]. Low concentrations of BPA induce early vaginal opening and disruption of the menstrual cycle by altering hormone synthesis mechanisms such as ones that decrease the serum levels of luteinizing hormone [80]. In addition, BPA can be transferred to the foetuses from the placenta and umbilical cord blood in pregnant mice, which alters postnatal development and sexual maturity even with low concentration doses of BPA [81].

#### Nonyl-phenol and octyl-phenol

Alkylphenols are present in household detergents and insecticides [81]. Alkylphenols can bind to the ERα and induce *vitellogenin* gene expression in animals [83]. Recently, *in vitro* study have shown that alkylphenol compounds, such as OP and NP, are very potent oestrogenic agents, and the binding affinity ERs to OP is approximately 1000-fold less than that of oestrogen [84]. NP and OP bind to the ER-α and induce ER-α-dependent gene transcription through the ERE promoter in yeast and mammalian cells [85]. Alkylphenols also stimulate the proliferation in MCF-7 cells similar to E2. The oestrogenicity of NP has been evaluated in a mouse uterotrophic bioassay which showed that high doses of NP accelerates vaginal opening with a longer oestrous cycle in a three-generation study of rats exposed to NP. NP can also be detrimental to steroidogenesis and disrupts endocrine systems through ER pathways, which induces apoptosis in primary germ and Sertoli cells [86]. Exposure of male mice to NP causes severe testicular abnormalities including decreased testis growth, inhibited immature germ cell differentiation and reduced sperm counts [87].

#### Methoxychlor

Methoxychlor is a pesticide developed as a substitute for DDT. Although MXC has a similar structure to that of DDT, it is more rapidly metabolized and does not accumulate or concentrate in adipose tissue in mammals including human beings. MXC has weak oestrogenic activity and binds to both the ER-α and ER-β. Similar to E2, MXC increases uterine weight in ovariectomized rats, and can exert adverse developmental and reproductive effects on laboratory animals [88]. However, there are differences between the *in vivo* activities of MXC and E2. For example, MXC does not increase FSH or LH levels in rats unlike E2. Moreover, MXC acts as an ER agonist in the uterus and an ER antagonist in the ovary. An exposure to MXC affected normal ovarian function *via* alteration of DNA methylation [89]. Bisphenolic MXC is a metabolite of HPTE, which is considered to have a 100-fold higher affinity for the ER-α than that observed for MXC [90]. HPTE is a potent ER-α agonist but a weak ER-β and AR antagonist. HPTE also reduces testosterone levels both *in vitro* and *in vivo* because of its weak AR antagonist activity. Exposure to HPTE can result in neurological and hormonal abnormalities which alter reproductive organ morphology and hormonal cycles [91].

#### Endosulfan

Endosulfan is a well-known insecticide used to eradicate insects for agriculture and wood preservation [92]. Residual endosulfan on crops can usually be broken down within a few weeks but can persist for a few years in soil. The routes of endosulfan exposure for human beings is usually *via* the consumption of food containing endosulfan and skin contact with contaminated soil [93]. However, laboratory animal studies suggest that continual long-term exposure of endosulfan damages the kidneys, central nervous and immune system [94, 95]. Therefore, a prolonged exposure to endosulfan may induce neurological symptoms and toxicity in mammals [96, 97].

#### Phyto-oestrogens

Phyto-oestrogens are non-steroidal polyphenolic compounds found in some plants including soybeans, and can act as steroid hormones in animals and human beings [98]. Diets rich in phyto-oestrogens may exert protective effects against oestrogen-related diseases such as ovarian and breast cancer in women although it is not clear if these anticancer effects are directly owing to the phyto-oestrogen-associated oestrogenicity [99]. Isoflavonoids are the most extensively studied class of phyto-oestrogens. Soybeans contain high concentrations of isoflavonoids. Genistein and other isoflavonoids are frequently found in the human diet. Metabolites of genistein are ER agonists with relatively low potency. Genistein can nevertheless compete for ER binding because of its structural similarity with endogenous oestrogen, and can have agonistic and/or antagonistic effects [100]. However, genistein has a lower affinity than E2, and preferentially binds to the ER-β rather than the ER-α [101].

Genistein inhibits cell proliferation in breast and prostate cancer *in vivo* and *in vitro*, and controls gene expression which is critical for cell cycle transition, apoptosis and signal transduction. This compound can thus stimulate apoptosis and inhibits the activation of important signalling events related to cell survival and apoptosis such as Akt and NF-κB signalling [56].

As mentioned previously, EDCs are present at low levels in the environment compared with experimental doses. Recent studies have reported that exposure to a combination of oestrogenic chemicals causes synergistic results, and these effects are of global concern [102]. Exposure to various EDCs mixtures may primarily induce additive responses through different complex pathways, which can be predicted that synergistic interaction of EDCs mixture make foetal results in human health.

### Effects of EDCs on human health

Various EDCs have adversely influenced on human health, resulting in disruption of reproductive development and function, stimulation of cancer growth, neuronal and immune system dysfunction in the body [103].

#### Disruption of reproductive development and function

Endocrine-disrupting chemicals are associated with abnormalities of the reproductive system in wildlife and laboratory animals [8]. However, the impact of environmentally relevant, low level exposure of these materials on human beings is still unknown. Development and maturation of female reproductive glands and tract are completed during the somatological process [104, 105]. If this process is altered by endogenous and/or exogenous factors during key periods, the reproductive system could be unexpectedly affected for multiple generations [106]. A normal function of human ovaries is related with successful differentiation of germ cells into oocytes, which is sensitive to some reagents such as EDCs. There is extensive evidence for the effects of BPA and OP on the development of the uterus and mammary glands [2]. Studies of both *in vivo* rats and mice have shown that continual exposure to BPA resulted in a massive endometrial surface and changes in vaginal opening timing and cytology [107].

The male reproductive system can also be disrupted by the effects of individual or mixtures of EDCs [108]. Endocrine-disrupting chemicals are thought to increase the frequency of severe abnormalities such as testicular cell cancer, semen quality reduction and pathological manifestation of urogenital disorders including hypospadias and cryptorchidis [109]. In male rodents, these abnormal symptoms can be observed after foetal exposure to phthalates and BPA. Several studies have suggested that disorders in both female and male reproductive function are linked to EDCs exposure [108, 110]. As previously mentioned, foetal exposure to EDCs may have effects on reproductive capabilities in human beings.

#### Stimulation of cancer growth by EDCs

Cancer is significantly increasing in frequency among industrialized nations. This disease is caused by cell cycle dysregulation and changes in cell cycle-related gene expression levels. Cyclin D1 overexpression is associated with increased expression of CDK4 in most types of cancer cells [111]. BPA and E2 treatment results in elevated expression of cyclin D1 and CDK4 in a relatively high percentage of breast cancer and endometrial cancer cells thereby promoting G1, S and G2-M phase transition [112]. Endocrine-disrupting chemicals are able to induce the proliferation of MCF-7 and inhibit apoptosis of cancer cells as a result of cell cycle dysregulation [113]. Human mammary glands undergo postnatal programmed architectural changes that occur in response to endogenous hormone signalling. Many studies of endocrine disruptors have confirmed that frequent exposure to EDCs can interrupt normal tissue organization and the interaction between stromal and epithelial layers of organs [114]. Endocrine-disrupting chemicals enhance the risk for the progression of neoplastic lesions to cancer in mammary glands and ovaries *via* their detrimental effects on important regulatory mechanisms such as organization of reproductive tissue [115].

Prostate cancer is a common cancer in males with a poor diagnosis, and steroid hormonal signalling appears to play a critical role in its formation and metastasis [116]. The prostate expresses both ER-α and ER-β, and steroid hormone-mediated signalling regulates the development of male reproductive organs and sexual characteristics in adulthood [117]. Moreover, prostate gland cells are particularly sensitive to oestrogenic responses during the developmental and adolescent periods [118]. Although it is difficult to study the direct association between prostate cancer risk and EDC exposure in human beings [119], prostate cancer cell proliferation is stimulated by EDCs treatment in animal models [120].

Endocrine-disrupting chemicals, including BPA, can promote the growth of neuroblastoma to a level similar to that of E2 [121]. Most neuroblastoma tumour cells are involved with high vascular endothelial growth factor (VEGF) expression [122], a key growth factor in tumour angiogenesis, resulting in both disease progression and poor prognosis [123]. However, BPA may promote neuroblastoma growth by modulating VEGF production in xenograft models. The results suggest that BPA promotes angiogenesis *via* its effects on growth factor expression in neuroblastoma cells [124].

In summary, environmental endocrine disruptors potently stimulate the proliferation of cancer cells both *in vitro* and *in vivo* [125]. It is possible that EDCs can interfere with metabolism and hormonal balance. These compounds may also affect cell cycle regulation and ER-dependent pathways during carcinogenesis [112, 126].

#### Neuronal and immune system dysfunction

Neuroendocrine systems are critical for the control of homeostasis and physiologic development. The physiological mechanism controlled by neuroendocrine systems is highly complex, these process make a successful organization of the hypothalamus and the pituitary gland in brain [127]. For this organization, several specific hormones and proteins need to be produced and regulated in a timely manner to maintain homeostasis [128]. Furthermore, these neuroendocrine systems control various important functions such as reproduction, stress responses, growth, lactation, metabolism, energy balance [129, 130] and other processes which mediate the ability of an organism to respond to its environment through rapid and sustained responses [127].

Endocrine-disrupting chemicals can exert neurobiological and neurotoxic effects. These chemicals may act on nuclear hormone receptors expressed in cells in hypothalamus, pituitary gland and other areas of the brain [127]. The neuroendocrine effects of EDCs may occur *via* numerous neurotransmitters such as dopamine and noradrenaline which are sensitive to endocrine disruption [128]. These findings appear to demonstrate the neurological effects of EDCs on cognition, memory and reproductive behaviours. For example, a previous study showed that decreased dopamine concentrations in the brain are a consequence of PCB exposure because this can inhibit dopamine synthesis and change the sensitivity of receptors in cholinergic synapses [130]. Even though it is difficult to precisely assess the neuroendocrine effects of EDCs owing to the complex of nature the involved physiological systems, studies of these effects in rodents and human beings have to take persistently in consideration [131].

In general, sex hormones such as testosterone help stimulate the immune system [132]. This logically implies that immune system is sensitive to EDCs in a manner similar to that of endogenous hormones. However, synthetic non-steroidal compounds such as DES are potent suppressors of thymus-dependent cellular immune responses *via* gene expression alterations in animal [133]. Susceptibility of the immune system to toxic chemicals is increased during the perinatal period as shown by *in vivo* studies of various compounds such as dioxin [134].

### Novel assays to evaluate EDCs

There is growing concern about the increasing health problems posed by EDCs in the environment that can impact human endocrine and reproductive systems. However, there is no standard method to determine whether an environmental chemical is an EDC or to measure its potency [135]. Thus, efficient and precise assays are required to evaluate EDCs potency and understand their mechanisms of action. These can be used to examine the detrimental effects of EDCs on human beings and wildlife. As many EDCs are thought to impact sex hormone functions, the findings of laboratory animal studies are potential evidence of endocrine disruption that can contribute to human health problems. *In vitro* methods for assessing oestrogenic compounds have been developed including yeast oestrogenic screening, ER binding, MCF-7 human ER-positive breast cancer cell and ERE-luciferase activity assays [75]. Other recent studies indicated that weak oestrogenic alkylphenols including BPA activate the transcription of cAMP-responsive element binding protein (CREB) and phosphorylation of CREB *via* a non-classical membrane ER in a calcium-dependent manner [136]. Furthermore, several biomarkers in *in vitro* models including cell-based endogenous genes such as those that encode pS2, mucin 1, ornithine, steroid hormone receptors (ERs, ARs and PRs) and vitellogenin [137, 138]. However, expression levels of almost all these genes are too low to be detected for evaluating the effects of EDCs at environmentally relevant concentrations.

Although oestrogen-binding affinity in mammalian cell lines and yeast screening assays have been extensively used to determine the oestrogenicity of xenoestrogen compounds, these assays cannot account for the biological effects of a compound on metabolism [139]. Uterotrophic activity and vaginal cornification assays have been traditionally used as *in vivo* methods for examining the ability of oestrogenic compounds to change the uterine wet weight or extent of vaginal cornification after treatment with the suspected EDCs [140]. However, uterotropic activity does not always correlate with the oestrogenic activity of chemicals. An *in vivo* approach for investigating responses of ERE promoters using transgenic animals expressing ERE-luciferase constructs has been reported. This method does not identify specific genes that are activated or repressed by ER–ERE interactions [141]. However, *In vivo* assay to assess oestrogenicity of EDCs is controversial and not clear.

A further study is required to develop ideal screening methods to determine EDCs potency at an environmentally persistent concentration with cost-effective and timely manner. Recent studies have been conducted to detect oestrogenic disrupting chemicals in rats [142]; the results indicated that the both mRNA and protein expression levels of calbindin-D_9k_ (CaBP-9k) can be used as a novel biomarker for identifying EDCs [143]. CaBP-9k is a 9 kD cytoplasmic protein with high binding affinity for calcium and belongs to the intracellular protein family. It has been postulated that CaBP-9k may be associated with the regulation of myometrial activity that controls calcium levels in the cell [144]. CaBP-9k gene expression is very sensitive to oestrogenic activity of EDCs, which gives a possibility to detect the oestrogen activity of EDCs by its enhanced gene expression [21]. To understand the underlying hormonal mechanism, studies of CaBP-9k gene expression have been experimented in rats [145]. Oestrogen is known to induce up-regulation of CaBP-9k gene expression whereas progesterone is down-regulated in rat uterus during early pregnancy and the oestrous cycle [146, 147]. In addition, the effects of alkylphenols such as BPA, OP and NP as well as E2 can increase CaBP-9k mRNA and protein levels through a dose- and time-dependent manner at rat pituitary GH3 cells [148]. CaBP-9k assays for studying immature rat uterus are very useful and sensitive, and can identify the oestrogenic or progestogenic activity of EDCs [21, 149, 150].

## Conclusion

Unlike oestrogen, EDCs can lead to adverse biological effects in animals and human beings *via* hormone receptor binding. These findings suggest that biochemical pathways associated with EDCs may involve the ER-dependent signalling pathway. Exposure to these chemical has detrimental effects on metabolism along with endocrine and reproductive systems that can persist for multiple generations. In addition, EDCs may stimulate carcinogenesis and potentially alter neuronal and immune systems. Thus, more sensitive and accurate *in vitro* and *in vivo* strategies are necessary for detecting the adverse actions and effects of EDCs. Understanding of the exact mechanism underlying the effects of these compounds is required for promoting human health. In particular, the impact of combinations of EDCs must be understood that they are generally released into environment as mixtures rather than individual reagents. The adverse results, caused by exposure to many EDCs, strongly impact the endocrine system, metabolism, homeostasis and reproduction. Therefore, it is needed to disclose the mechanism of EDCs action in organs and important to evaluate the synergistic effects of exposure to multiple EDCs in future studies.
